# Variants in *CLCN1* and *PDE4C* Associated with Muscle Hypertrophy, Dysphagia, and Gait Abnormalities in Young French Bulldogs

**DOI:** 10.3390/ani14050722

**Published:** 2024-02-25

**Authors:** G. Diane Shelton, James R. Mickelson, Steven G. Friedenberg, Jonah N. Cullen, Karina Graham, Missy C. Carpentier, Ling T. Guo, Katie M. Minor

**Affiliations:** 1 Department of Pathology, School of Medicine, University of California San Diego, La Jolla, CA 92093, USA; liguo@health.ucsd.edu; 2 Department of Veterinary and Biomedical Sciences, College of Veterinary Medicine, University of Minnesota, Saint Paul, MN 55108, USA; micke001@umn.edu; 3 Department of Veterinary Clinical Sciences, College of Veterinary Medicine, University of Minnesota, Saint Paul, MN 55108, USA; fried255@umn.edu (S.G.F.); cull0084@umn.edu (J.N.C.); minork@umn.edu (K.M.M.); 4 Veterinary Specialists of Sydney, Sydney, NSW 2228, Australia; karina@vsos.com.au; 5 Animal Emergency & Referral Center of Minnesota, Oakdale, MN 55128, USA; carpentier@mnveterinaryneurology.com

**Keywords:** myopathy, dog, whole genome sequencing, Sanger sequencing, non-dystrophic myotonia

## Abstract

**Simple Summary:**

Muscle hypertrophy, swallowing disorders, and gait abnormalities are clinical signs common to many muscle diseases, including muscular dystrophies, non-dystrophic myotonias, myopathies associated with deficiency of myostatin, and acquired inflammatory myopathies. Here we describe four clinical cases in young French bulldogs displaying the above clinical signs. Using whole genome sequencing in two of the cases and Sanger sequencing validation, variants were identified in the chloride channel gene *CLCN1*, which causes non-dystrophic congenital myotonia, and in the phosphodiesterase gene *PDE4C*, which is the major phosphodiesterase expressed in skeletal muscle and may play a role in decreasing muscle atrophy. In one case for which whole genome sequencing was not performed, genotyping for the identified variants was not confirmed. In the remaining case, the *CLCN1* variant was confirmed with genotyping. Identification of variants in genes associated with this clinical presentation may assist breeders with breeding programs designed to eliminate them in this currently very popular breed. Further, identification of new disease-causing variants may help us to identify therapies to reverse muscle atrophy in individuals affected by this group of neuromuscular diseases.

**Abstract:**

(1) Background: Muscle hypertrophy, swallowing disorders, and gait abnormalities are clinical signs common to many muscle diseases, including muscular dystrophies, non-dystrophic myotonias, genetic myopathies associated with deficiency of myostatin, and acquired inflammatory myopathies. Here, we investigated underlying causes of this triad of clinical signs in four young French bulldogs via muscle histopathology coupled with whole genome and Sanger sequencing. (2) Methods: Dogs were evaluated by veterinary clinical internists and neurologists, and biopsies were obtained for histopathological diagnosis. DNA was submitted for whole genome sequencing, followed by bioinformatics evaluation and confirmation of variants via Sanger sequencing in two cases. (3) Results: Two novel variants were identified. The first, found in two related French bulldogs, was a homozygous variant in the chloride channel gene *CLCN1* known to cause non-dystrophic congenital myotonia, and the second, found in an unrelated French bulldog, was a heterozygous variant in the cAMP phosphodiesterase gene *PDE4C*, which is the major phosphodiesterase expressed in skeletal muscle and may play a role in decreasing muscle atrophy. An underlying molecular basis in one other case has not yet been identified. (4) Conclusions: Here, we identified two novel variants, one in the *CLCN1* and one in the *PDE4C* gene, associated with clinical signs of muscle hypertrophy, dysphagia, and gait abnormalities, and we suggested other bases of these phenotypes in French bulldogs that are yet to be discovered. Identification of genes and deleterious variants associated with these clinical signs may assist breeders in improving the overall health of this very popular breed and may lead to the identification of new therapies to reverse muscle atrophy in people and animals with neuromuscular diseases.

## 1. Introduction

Dysphagia, muscle hypertrophy, and gait abnormalities are clinical signs that may be associated with diverse neuromuscular diseases, including but not limited to inflammatory myopathies [1,2,3,4], muscular dystrophies [5,6,7], non-dystrophic myotonias [8,9,10,11,12], and congenital myopathies associated with myostatin deficiency [13,14]. Specific variants have been identified in the dystrophin gene [15], sarcoglycan genes [15,16], the chloride channel gene [8,9,10,11,12], and the myostatin gene [13,14] that result in localized or generalized muscle hypertrophy with or without dysphagia. In these cases, it can be difficult if not impossible to reach a diagnosis based on clinical presentation alone, and diagnostic procedures, including electrodiagnostic examination (electromyography, measurement of motor and sensory nerve conduction velocities), examination of muscle biopsies, and immunostaining for protein localization, are required to reach a diagnosis. For breeding animals, identification of a specific gene variant via whole genome sequencing (WGS) and bioinformatics is an increasingly useful tool to manage or even eliminate a damaging variant from the breeding population. In some cases, identification of the gene variant can be helpful in clarification of the underlying disease when histopathological evaluation cannot be specific. For example, recently, a 3-month-old male intact French bulldog was reported with clinical signs of dysphagia and dyspnea from severe macroglossia, low head carriage, and tetraparesis. Using WGS, a *DMD* p.F1125* frameshift/stop-gain variant was identified, confirming the dog had X-linked dystrophin-deficient muscular dystrophy [15].

Here, we described a new *CLCN1* variant in two young French bulldogs with dysphagia and increased muscle mass in the shoulder and neck as the primary presenting clinical signs. In addition, we described a *PDE4C* variant in a fourteen-month-old female spayed French bulldog that was evaluated for a chronic history of thoracic limb stiffness and gross hypertrophy of the proximal thoracic limb muscles. We proposed that these *CLCN1* and *PDE4C* variants may be causative for the clinical signs, added to the catalog of likely *CLCN1* disease-causing variants in dogs, and proposed that the *PDE4* variant leads to consequent elevation of cellular cAMP levels that results in increased muscle mass.

## 2. Materials and Methods

### 2.1. Animals

Dogs included in this study were clinical cases evaluated by specialists in veterinary internal medicine or neurology, and tissues were collected for diagnostic purposes with owner consent.

Case 1. A 24-week-old female French bulldog was evaluated at the Veterinary Specialist and Emergency Center in Sydney, Australia, for intermittent dysphagia and upper airway obstruction. The dog was obtained from a breeder by the owner at 12 weeks of age and chosen because it was smaller in size than the unaffected littermates. A palatoplasty was performed at another veterinary hospital, but the dog did not improve and was referred for further evaluation. A sibling had a similar dysphagia and upper airway disease and was euthanized the previous week.

Case 2. A 22-week-old female French bulldog obtained from the same breeder as Case 1 and with the same father was evaluated at the Veterinary Specialist and Emergency Center in Sydney, Australia. The owner obtained the dog at 10 weeks of age, and since that time, the dog had shown excessive swallowing and choking episodes. In one choking episode, the dog became stiff and fell down the stairs. The dog was referred for evaluation of a swallowing disorder.

Case 3. A 14-month-old female spayed French bulldog was presented to the Animal Emergency and Referral Center, Oakdale, MN, USA, with a chronic history of pelvic limb weakness and thoracic limb muscle hypertrophy. The dog had a stiff thoracic limb gait with the inability to extend the thoracic limbs when walking (Supplemental Video S1) and had difficulty righting itself when laying on its back. The dog was obtained from the breeder at approximately 1 year of age, was the runt of the litter, and was presented for neurologic evaluation.

Case 4. A 3-month-old male French bulldog was obtained from a breeder. The dog had been hospitalized with Parvovirus, and by 4–5 months of age, it had difficulty with ambulation. It had undergone upper airway surgery (soft palate resection, laryngeal sacculectomy, nares resection) and was treated for allergic dermatitis, but continued to have a short-stride, stiff, stilted gait and extremely large appendicular muscles (Supplemental Video S2). Prior to referral to the sports medicine specialist at 1 year of age, the dog was evaluated by a board-certified neurologist at the Central Hospital for Veterinary Medicine, North Heaven, CT, USA, and joint taps, MRI, and CSF analyses had been performed, with all results normal. As of this writing, the dog is still alive and maintains a stiff, stilted gait.

### 2.2. Light Microscopy, Immunofluorescent Staining, and DNA Extraction

Unfixed chilled and formalin-fixed diagnostic muscle specimens were collected post-mortem from Cases 1 and 2, including the diaphragm, tongue, supraspinatus, epaxial, masseter, and quadriceps muscles. A diagnostic muscle biopsy specimen from the triceps muscle of Case 3 was collected under general anesthesia. Muscle samples were shipped via an express service under refrigeration to the Comparative Neuromuscular Laboratory, University of California San Diego. The unfixed muscle specimens were subsequently flash-frozen in isopentane pre-cooled in liquid nitrogen, followed by storage at −80 °C until further processed. Following sectioning, cryosections were evaluated using a standard panel of histochemical stains and reactions [17]. As a presumptive diagnosis of a form of muscular dystrophy was suspected by the clinicians, additional cryosections were cut and stained for indirect immunofluorescence, as previously described [18]. Monoclonal antibodies against the rod (1:100, NCL-DYS1) and carboxy-terminus (1:100, NCL-DYS2) of dystrophin, utrophin (1:20, NCL-DRP2), and developmental myosin heavy chain for regenerating fibers (1:20, NCL-dMHC) were all obtained from Novocastra Laboratories, Newcastle, UK. A monoclonal antibody against caveolin 3 (1:100) was obtained from Santa Cruz Biotechnology Inc, Dallas, TX 75220, USA. Polyclonal antibodies against laminin α2 (1:200), α-sarcoglycan (1:200), and collagen VI (direct apply, monoclonal antibody 3G7) were all gifts from Professor Eva Engvall [19,20]. Genomic DNA from Cases 1–3 was isolated from archived frozen diagnostic muscle biopsy specimens using the Qiagen DNEasy kit following package instructions. A cheek swab was collected from Case 4, and genomic DNA was isolated using the Qiagen Puregene kit following package instructions. Genomic DNA was isolated from EDTA whole blood on 2 unaffected French bulldogs related to Cases 1 and 2 using the DNEasy kit following package instructions.

### 2.3. Whole Genome Sequencing and Variant Analysis

A PCR-free library was prepared from Cases 1 and 3, and 150 bp paired-end reads were generated on an Illumina HiSeq 4000 sequencer using GeneWiz (South Plainfield, NJ 07080, USA). For Case 1, 819 million paired-end reads were generated, corresponding to a mean 47-fold genome-wide coverage; for Case 3, 348 million paired-end reads were generated, corresponding to a mean 21-fold genome-wide coverage. Reads were mapped against the dog reference genome assembly (CanFam4) and processed using the OnlyWAG pipeline as described [21]. Raw sequence reads are available in NCBI’s Short Read Archive at SRR27051485 and SRR27051486 as part of BioProject PRJNA937381 with the IDs D08521 and D08161, respectively [22].

WGS variants from Cases 1 and 3 were compared to those of control genomes from an internal WGS database developed at the University of Minnesota containing a total of 671 dogs of 62 diverse breeds (including 9 mixed breeds and 21 additional French bulldogs from unrelated projects). The WGS data from these 671 dogs were processed using the same bioinformatics pipeline referenced above. We searched for variants that were only present in the affected dogs and that were within or near coding exons. We then prioritized these variants for further evaluation based upon their likely effect on the encoded protein as identified via Variant Effect Predictor [23] using the following categories: high impact (frame shift, loss or gain of stop or start codon, affecting a splice junction), moderate impact (missense), or low impact (synonymous, near splice junction). We also cross-referenced the function of the protein encoded by the gene, for which the variant was identified using publicly available resources (e.g., PubMed, GeneCards [24]), and prioritized variants in genes known to be associated with muscle function. A list of the unique coding variants of Cases 1 and 3 is provided in Table S1.

### 2.4. Sanger Sequencing

Sanger sequencing of a PCR amplicon was employed for genotype Cases 2 and 4 for the *CLCN1* variant found in Case 1, as well as for the *PDE4C* variant identified in Case 3. PCR utilized standard conditions with the primers shown in Table 1.

## 3. Results

### 3.1. Animals and Histopathology

**Figure 1:**
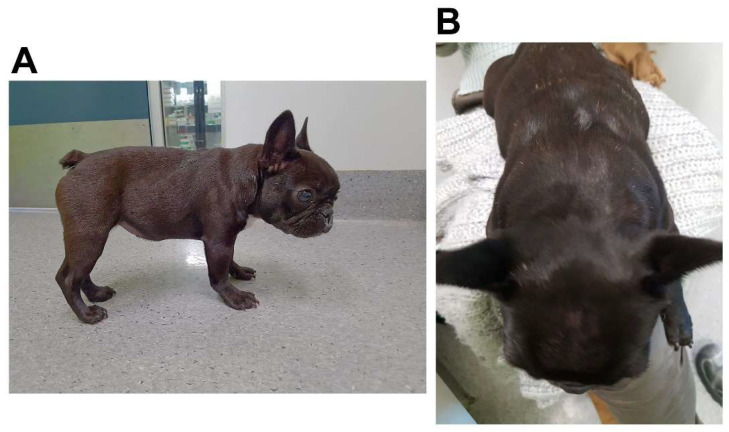
A 24-week-old female French bulldog (Case 1) was evaluated for intermittent dysphagia and upper airway obstruction. Neck musculature was thickened, as shown in a lateral view (**A**) and a dorsal view (**B**), as were muscles of the proximal thoracic limb. The clinical phenotype was similar in the related French bulldog (Case 2).

**Figure 2:**
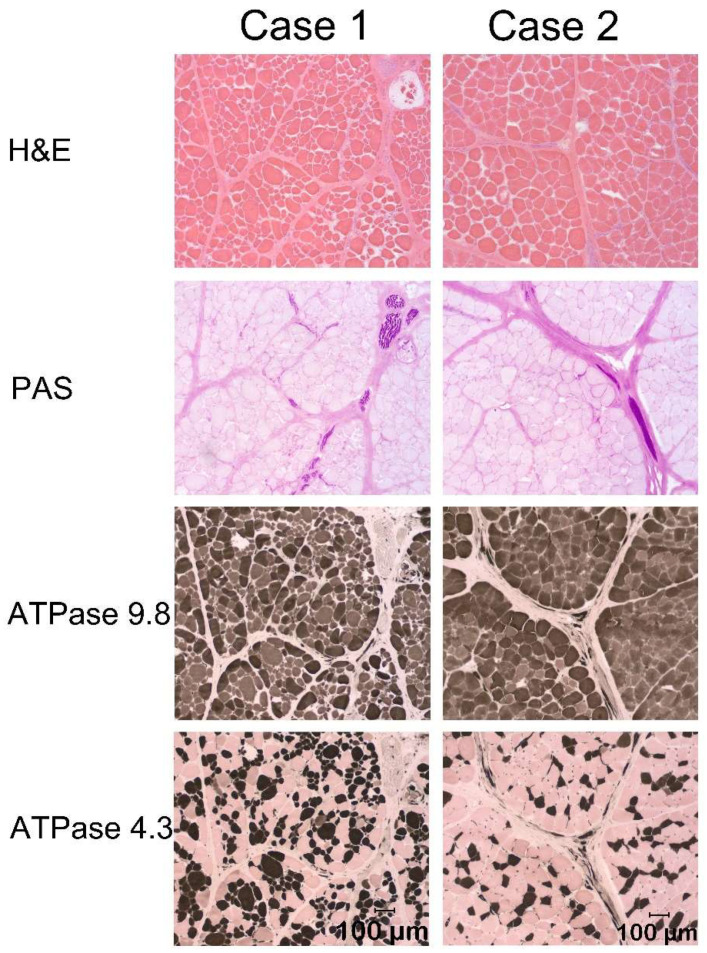
Representative cryosections from the epaxial muscles of Cases 1 and 2 showed minimal changes, including variability in myofiber size (H&E stain). Intramuscular nerve branches were normal in appearance (dark purple stained structures, PAS stain). A normal mosaic pattern of muscle fiber types was present in both cases without obvious fiber type grouping (ATPase reaction at pH 9.8 and 4.3). Bar in bottom image of each case = 100 µm for each image.

Case 1. On physical examination, the dog was approximately two thirds the size of the siblings. The neck and shoulder musculature were very prominent and thickened (Figure 1). The ventral tongue could be palpated readily in the submandibular space and was firm and thick. Creatine kinase (CK) activity was mildly elevated at 1213 IU/L (reference < 401). An esophageal contrast study was evaluated under fluoroscopy and showed marked cricopharyngeal dysphagia. Tongue cyanosis was noted during this phase. Since a diagnosis of muscular dystrophy was suspected, which carries a poor prognosis, the dog was euthanized, and post-mortem muscle samples were collected from the masseter, temporalis, tongue, quadriceps, and cervical epaxial muscles. Histopathology in muscle cryosections showed minimal changes with variability in myofiber size and retention of a normal mosaic pattern of muscle fiber types (Figure 2). Immunofluorescence staining of muscle cryosections using antibodies against dystrophy-associated proteins did not reveal any abnormal staining patterns compared to control muscle.

Case 2. On physical examination, the dog was able to stand and walk but was generally thin. A muscular neck and firm, fleshy, and symmetrical thickening in the intermandibular region and macroglossia were noted. CK activity was mildly elevated at 706 IU/L (reference < 401). A diagnosis of cricopharyngeal dysphagia was made, and an esophageal contrast study was performed with fluoroscopy. Laryngeal function was evaluated under sedation. Severe cricopharyngeal dysphagia and severe laryngeal collapse were diagnosed, and the owner elected euthanasia. As a form of muscular dystrophy was suspected, muscle specimens were collected post-mortem from the supraspinatus, epaxial, masseter, quadriceps, diaphragm, and tongue (Figure 2). Immunofluorescence staining of muscle cryosections using antibodies against dystrophy-associated proteins did not reveal any abnormal staining patterns compared to control muscle. 

Case 3. On physical examination, the dog was bright and alert with normal vital parameters and normal thoracic auscultation. Neurological examination showed the dog was ambulatory paraparetic, with an inability to fully extend the thoracic limbs due to marked hypertrophy of the supraspinatus, infraspinatus, biceps, and triceps muscles bilaterally. Marked hypertrophy of the temporalis muscle was also noted. Conscious proprioception, segmental reflexes and cranial nerves were intact. A multifocal etiology was suspected, with concern for a neuromuscular disease affecting the thoracic limbs and temporalis muscles and a T3-L3 myelopathy affecting the pelvic limbs. Pre-anesthetic blood work was unremarkable, including a normal serum CK activity of 113 IU/L (reference 10–200). A full neuroaxis MRI was performed that showed a subarachnoid diverticulum extending from T12 through the L1-2 disk space. Syringohydromyelia was also present from C2 through C4. As a neuromuscular disease was suspected of affecting the thoracic limbs, electromyography and muscle biopsies were performed under general anesthesia.

**Figure 3:**
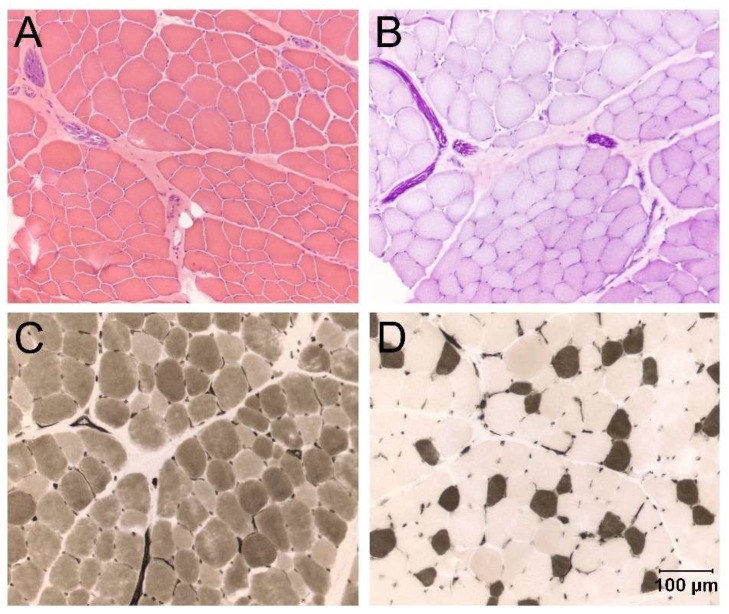
Cryosections from the left triceps muscles of Case 3 showing variability in myofiber size without other specific cytoarchitectural abnormalities ((**A**), H&E stain). Intramuscular nerve branches were normal in appearance ((**B**), PAS stain, intramuscular nerve branches are dark purple structures). Fiber typing using the myofibrillar ATPase reaction at pH 9.8 (**C**) and pH 4.3 (**D**) showed a normal mosaic pattern of muscle fiber types without obvious fiber type grouping or fiber type predominance. Bar in D lower right corner = 100 μm for all images.

Electromyography was performed and showed fibrillation potentials and positive sharp waves in all muscles examined, including the supraspinatus, infraspinatus, biceps, and triceps muscles. Histopathologic examination of cryosections from the left triceps muscle (Figure 3) showed variability in myofiber size, with fiber diameters ranging from 14 to 100 µm. No inflammatory or degenerative changes were identified. A non-inflammatory, possibly congenital myopathy was suspected.

Case 4. On presentation to the sports medicine specialist, the appendicular muscles were extremely large and firm even at rest, with increased tone and tension, particularly in the thoracic limb extensors, triceps, gastrocnemius, and hamstring muscles. The gait was short-strided with stiffened toe walking on all limbs. The dog would ambulate with great difficulty and preferred to crawl. The appendicular joints (elbow, stifle, carpi, tarsi) were difficult to flex. The CK activity was mildly elevated at 960 IU/L (reference 59-895 IU/L). A congenital neuromuscular disease was suspected, and cheek swabs were collected for DNA genotyping.

### 3.2. Whole Genome Sequencing and Variant Analysis

**Figure 4:**
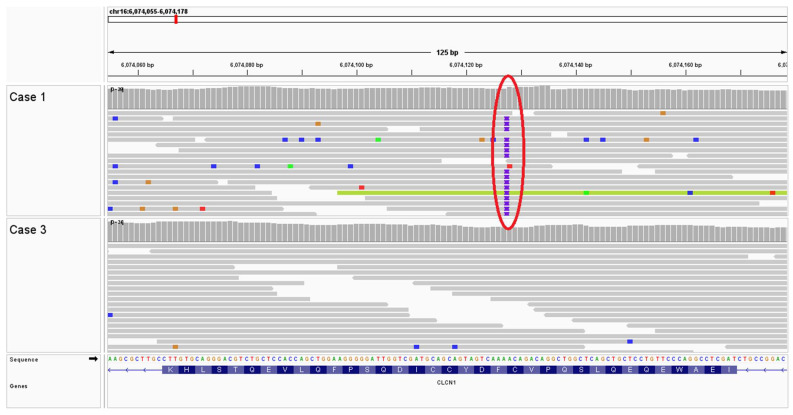
Integrative Genomics Viewer (IGV) view of an 8 bp insertion in *CLCN1* exon 21 (NP_001003124.1) in French bulldog Case 1 resulting in a p.F811Lfs*39 frameshift variant. The insertion, indicated by the purple “I” and surrounded by the red circle, is present in all but 1 variant-spanning read. This variant is absent in French bulldog Case 3.

**Figure 5:**
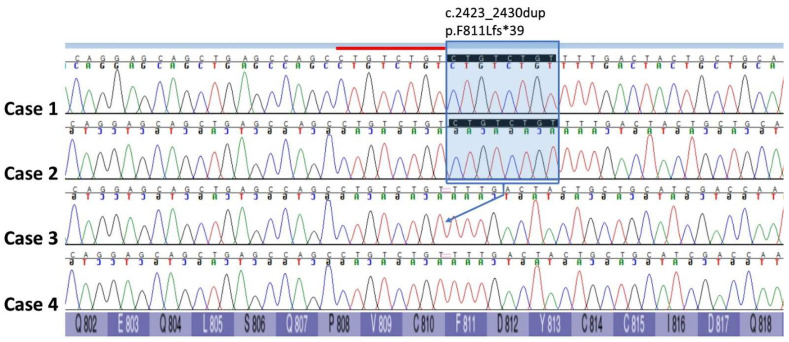
Sanger sequencing of all four French bulldog cases confirmed the homozygous *CLCN1* p.F811Lfs*39 variant in Case 1 and related Case 2. This variant was absent in French bulldog Cases 3 and 4. The insertion (blue boxed sequence) results from a duplication of the 8 base pairs under the red bar.

Cases 1 and 2. Following WGS and bioinformatic analysis of Case 1, review of the variant call format (VCF) file identified six homozygous and unique protein coding variants compared to the 671 genomes in the University of Minnesota WGS internal database. These variants included a homozygous 8 bp duplication insertion variant in the *CLCN1* gene resulting in a frameshift and premature stop codon (NP_001003124.1. p. F811Lfs*39). This variant was visually inspected with the Integrative Genomics Viewer (IGV) (Figure 4). Sanger sequencing of a PCR amplicon was employed to confirm this variant in Case 1, as well as to genotype Cases 2, 3, and 4. Case 2 was homozygous for the *CLCN1* variant found in Case 1 (Figure 5); the two unaffected French bulldogs related to Cases 1 and 2 were homozygous wild types.

**Figure 6:**
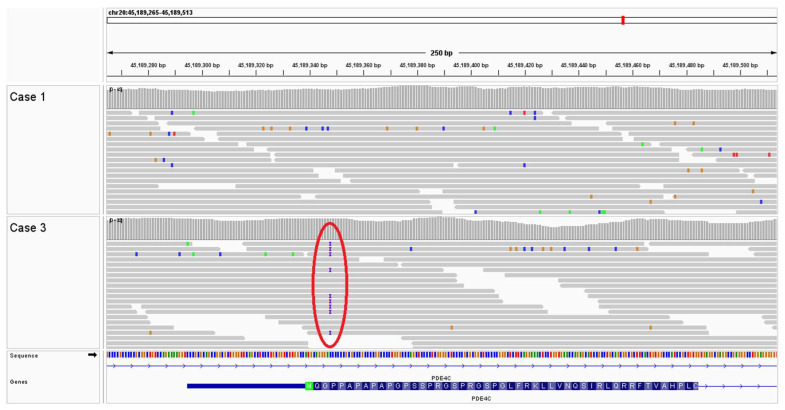
IGV view of a 1 bp “C” insertion in *PDE4C* (XP_038422764.1) in French bulldog Case 3 resulting in a p.A6Rfs*46 frameshift variant. The insertion, indicated by the purple “I” and surrounded by a red circle, was present in ~50% of variant-spanning reads. This variant was absent in French bulldog Case 1.

**Figure 7:**
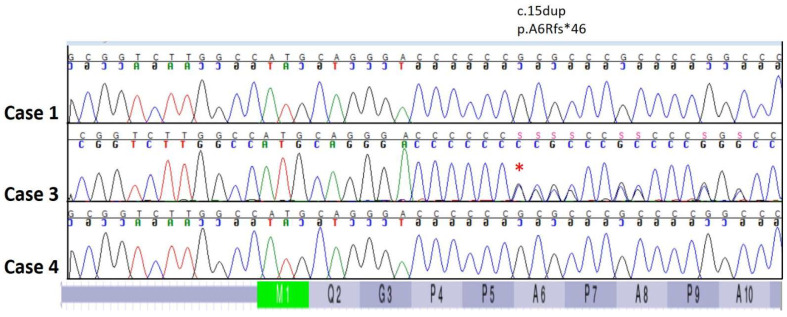
Sanger sequencing of French bulldog Cases 1, 3, and 4 confirmed the heterozygous *PDE4C* p.A6Rfs*46 variant in Case 3 indicated by the red (*). This variant was absent in *CLCN1* positive French bulldog Case 1 and unresolved in Case 4.

Cases 3 and 4. Following WGS and bioinformatic analysis of Case 3, review of the VCF file identified five homozygous and unique protein coding variants (one high, four moderate) compared to the 671 genomes in the University of Minnesota WGS internal database. Visual inspection of all five predicted variants indicated they were the result of mapping errors; consequently, thirty-one unique high impact heterozygous variants were next interrogated. A heterozygous 1 bp insertion in the *PDE4C* gene that results in a frameshift that knocks out almost the entire transcript (XP_038422764.1: p.A6Rfs*46) was identified in Case 3 (Figure 6). Sanger sequencing of a PCR amplicon containing the *PDE4C* variant identified in Case 3 was performed on Cases 1, 3, and 4 (Figure 7). Despite the similar clinical phenotype, breed, and age of onset, the *PDE4C* variant found in Case 3 was only present in Case 3 and not found in Case 4.

## 4. Discussion

French bulldogs have now become the number one breed registered by the American Kennel Club (https://www.akc.org/most-popular-breeds/; accessed on 14 February 2024). Expanding the spectrum of neuromuscular diseases affecting this breed and development of genetic testing to eliminate at-risk breeding animals is crucial. Here, we reported a novel homozygous variant in *CLCN1* in two related young French bulldogs from Australia and a heterozygous variant in *PDE4C* in an unrelated French bulldog from the USA that add to the list of genetic concerns that should be considered in designing breeding programs.

Mutations in the *CLCN1* gene encoding voltage-gated chloride channels in people with non-dystrophic myotonia congenita produce a wide spectrum of clinical phenotypes, including differences in age of onset and variability in affected muscles, severity of myotonia, and degree of muscle hypertrophy and muscle weakness [25]. To date, myotonia congenita in dogs has been associated with five different variants in the *CLCN1* gene (OMIA:000698-9615), including five different breeds [8,9,10,11,12]. The clinical presentation in our related French bulldogs with the novel *CLCN1* variant was primarily for oropharyngeal dysphagia, although muscle hypertrophy, most notably of the neck muscles and tongue, was described. Most previously reported cases of *CLCN1*-associated myotonia in dogs presented with stiffness that improved with activity and variable muscle hypertrophy [8,9,10,11,12]. Oropharyngeal dysphagia was described in the Labrador retriever, recently reported with a *CLCN1* variant [11]. In people, the non-dystrophic myotonias associated with mutations in the *CLCN1* gene produce a wide spectrum of clinical phenotypes with varying degrees of muscle hypertrophy and severity of myotonia [25]. Among the more than 200 *CLCN1* mutations that have been identified in people, not all have been phenotypically characterized. A broader spectrum of clinical presentations may also be found in dogs with *CLCN1* variants as more cases are investigated and variants are identified.

The young French bulldog with the heterozygous *PDE4C* variant (Case 3) presented for hypertrophy of the neck muscles and proximal thoracic limbs that resulted in a stiff gait and inability to flex the shoulder and elbow joints. The frameshift insertion identified in Case 3 knocks out almost the entire transcript (XP_038422764.1: p.A6Rfs*46); however, it must be recognized that this dog would still have a copy of the wild type gene. The clinical presentation in Case 4 was like that of Case 3, with marked muscle hypertrophy, most notably over the shoulders and thoracic limbs, as well as generalized stiffness and inability to flex the joints. Nevertheless, genotyping did not confirm the *PDE4C* variant in this dog, and a possible variant is still undetermined. It is possible that a different variant in the *CLCN1* or *PDE4C* genes or a variant in a different gene results in muscle hypertrophy and stiffness. Unfortunately, the molecular basis for the clinical signs in this young French bulldog remains unresolved.

The cAMP-selective phosphodiesterases (PDEs), by degrading the second messenger cAMP and lowering cellular cAMP levels, regulate and mediate several cellular responses to extracellular signals, including inhibition of cachexia associated with sepsis and neoplasia [26,27]. *PDE4* is the major PDE expressed in skeletal muscle [28,29]; however, not much is known about the physiological function of the four PDE4 subfamilies (A, B, C, and D). This is particularly true for PDE4C, due in part to its restricted distribution pattern and lack of research tools [30]. To the authors’ knowledge, *PDE4C* variants have not been associated clinically with muscle hypertrophy. However, there is evidence from mouse and rat models that phosphodiesterase 4B knockout prevents skeletal muscle atrophy resulting from denervation, limb immobilization, sepsis, or cancer [26,27]. Further, PDE4C inhibitors can reduce skeletal muscle atrophy in mice and rats following burn injury [27]. The usefulness of PDE4C inhibitors as a therapeutic target in a clinical setting to prevent or reverse muscle atrophy is worthy of further investigation. It remains to be determined whether the absence of PDE4C activity in skeletal muscle using PDE4C inhibitors can promote an increased muscle mass in the absence of an identifiable muscle disease or improve muscle atrophy in clinical neuropathies and myopathies. Histopathological evidence of an underlying muscle disease was not obvious in Case 3, although muscle mass was increased. We propose this novel heterozygous variant and the *PDE4C* gene as a potential cause of muscle hypertrophy, stiffness, and gait disorders.

The CK activity in all four French bulldogs was normal or only mildly elevated, making a diagnosis of a form of muscular dystrophy unlikely. The clinical presentation in the young male French bulldog previously reported with a variant in exon 25 of the *DMD* gene [15] included dysphagia and macroglossia, demonstrating some overlap with Cases 1 and 2. The CK activity, however, was markedly elevated in the dystrophic dog, and elevation of CK activity can help to differentiate a dystrophin-deficient muscular dystrophy from other early-onset inherited myopathies. The WGS ruled out a variant in the *DMD* gene in Case 1, and identification of the *CLCN1* variant in Case 2 made a concurrent variant in the *DMD* gene unlikely. Finally, immunofluorescence staining of muscle cryosections for dystrophy-associated proteins in both cases did not support a reduction in or absence of dystrophin.

There are a few shortcomings in this report. Electromyographic (EMG) studies to document myotonic discharges were not performed in Cases 1 and 2; however, clinical evidence of myotonia, including percussion dimpling and stiff gait, were not described. Phenotyping of the dam and sire was also not possible, although both French bulldogs diagnosed with the *CLCN1* variant were related and had the same clinical presentation. A further shortcoming of this study is that materials were not available to examine if levels of expression of the *PDE4C* gene or quantities of PDE4C protein were diminished, as might be expected for a heterozygote and a dominant pattern of inheritance. Further, we were unable to obtain DNA of the parents of Case 3 for genotyping or obtain EMG evaluations, muscle biopsies, and adequate DNA for WGS on Case 4. We also did not perform any functional studies to confirm deleterious effects of these mutations, nor did we attempt to determine the frequency of these mutations in the broader French bulldog population beyond their absence in the 21 French bulldogs in our WGS database, as these undertakings were beyond the scope of this case series. Despite this, we believe the comparative molecular and phenotypic evidence supports the *CLCN1* variant as causative and the *PDE4C* variant as possibly causative. They are worthy of further investigation to confirm their functional effects, determine their prevalence within the breed, and define optimal breeding strategies for French bulldog breeders.

## 5. Conclusions

Here, we reported a novel homozygous variant in *CLCN1* in two related young French bulldogs and a heterozygous variant in *PDE4C* in a young unrelated French bulldog associated with muscle hypertrophy, gait abnormalities, and dysphagia. A causative or possible variant has not yet been identified in a fourth young French bulldog with a similar clinical phenotype, suggesting other variants or genes are yet to be discovered. As more cases are characterized and variants are identified, the spectrum of clinical phenotypes and causative genes will likely expand and may assist breeders in elimination of disease-causing genes in designing a breeding program. Finally, identification of possible new disease-causing genes such as *PDE4C* may lead to the identification of new therapies to reverse muscle atrophy in people and animals with neuromuscular diseases.

## Figures and Tables

**Table 1:** Primer sequences used to genotype Cases 2 and 4 for the *CLCN1* and *PDE4C* variants.

Primers	Sequence	Size
*CLCN1* F	TATCCTGTGCTGCTCAAACG	564 bp
*CLCN1* R	GGAAGAGCTGGAAGACATGC	
*PDE4C* F	CTCAGTTTCCCCATTTGTCC	437 bp
*PDE4C* R	AAGAGAGGGAGGAGCAGAGC	

## Data Availability

Raw sequence reads are available in NCBI’s Short Read Archive at SRR27051786 and SRR27051485 as part of BioProject PRJNA937381.

## Supplementary Materials

The following supporting information can be downloaded at https://www.mdpi.com/article/10.3390/ani14050722/s1. Table S1: Unique coding variants for Cases 1 and 3; Video S1: Video showing muscle hypertrophy and stiff thoracic limb gait in Case 3 young French bulldog; Video S2: Video showing muscle hypertrophy and stiff gait in Case 4 young French bulldog.
